# Food insecurity in Detroit: exploring the relationship between patient-reported food insecurity and proximity to healthful grocery stores

**DOI:** 10.1017/S1368980021003128

**Published:** 2022-04

**Authors:** Sara Santarossa, Alex B Hill, Alexandra R Sitarik, Mackenzie Taylor, Susan Hawkins, Katherine Scher, Aaron Sohaski, Mohammed Baseer, Rachael Dombrowski, Alexander Plum, Christine LM Joseph

**Affiliations:** 1 Henry Ford Health System, Department of Public Health Sciences, 1 Ford Pl, Detroit, MI 48202, USA; 2 Wayne State University, Urban Studies and Planning and Detroit Food Map Initiative, Detroit, USA; 3 Henry Ford Health System, Population Health Management Department, Detroit, USA; 4 Wayne State University, Division of Kinesiology, Health and Sport Studies, Detroit, USA

**Keywords:** Food access, Food security, Geography, Healthcare, Social needs

## Abstract

**Objective:**

The objective of the current study was to determine if patients of a large health care system in Detroit who self-identify as food insecure live further away from healthy grocery stores compared with food secure patients. Second, we explored whether food insecurity and distance to healthy grocery stores are related to ecological measures of vehicle availability in the area of residence.

**Design:**

A secondary data analysis that uses baseline data from a pilot intervention/feasibility study.

**Setting:**

Detroit, Michigan, USA.

**Participants:**

Patients of Henry Ford Health System were screened for food insecurity to determine eligibility for a pilot intervention/feasibility study (i.e. Henry’s Groceries for Health), conducted through a collaboration with Gleaners Community Foodbank of Southeastern Michigan. Only patients residing in Detroit city limits (including Highland Park and Hamtramck) were included in the secondary analysis. Of the 1,100 patients included in the analysis, 336 (31 %) were food insecure.

**Results:**

After accounting for socio-demographic factors associated with food insecurity, we did not find evidence that food insecure patients lived further away from healthier grocery stores, nor was this modified by ecological measures of vehicle access. However, some neighbourhoods were identified as having a significantly higher risk of food insecurity.

**Conclusions:**

Food insecure patients in Detroit are perhaps limited by social and political determinants and not their immediate neighbourhood geography or physical access to healthy grocery stores. Future research should explore the complexity in linkages between household socio-economic factors, socio-cultural dynamics and the neighbourhood food environment.

Food security is an essential determinant of overall health and is categorised as high, marginal, low and very low^(1)^. Food insecurity can be defined as ‘a household-level economic and social condition of limited or uncertain access to adequate food’^(1)^. The above definition of food insecurity comprises what the USDA^(1)^ defines as ‘low’ (i.e. reports of reduced quality, variety or desirability of diet. Little or no indication of reduced food intake) and ‘very low’ (i.e. reports of multiple indications of disrupted eating patterns and reduced food intake). Previous literature extensively shows that people experiencing food insecurity generally exhibit less healthy eating behaviours, increased risk of obesity and related illnesses and increased risk of chronic disease^(2)^. As one in nine Americans are considered food insecure^(3)^, food insecurity has become the nation’s leading health and nutrition issue^(4)^.

Factors associated with food insecurity include unemployment, urban residence^(5)^, single-parent household^(6)^, low-income and race. The intersectionality of race/ethnicity and poverty accompanied by unjust social conditions and racial segregation further perpetrate inequities in availability of resources; for instance, spatially unjust food access. Thus, the relationship between being low income and a racial minority with food insecurity calls into question how these communities navigate their racialised food environment. Given that residential segregation is a component of structural racism, being a low-income racial minority in a low-resource neighbourhood calls into question how these communities navigate their food environment.

Food access can be compounded by several physical factors within a food environment, including transportation. Transport poverty is used in the literature to describe several forms of inequalities including transport affordability, mobility poverty and accessibility poverty, which may each relate and aid in explaining dimensions of food access^(7–9)^. Difficulty reaching food retail can be described as accessibility poverty, whereas transport affordability and mobility poverty (systemic lack of transport) are intertwined with food insecure populations as they are more likely to be low income. The literature suggests that transportation mode can drastically change levels of spatial accessibility to supermarkets^(10,11)^ as many people do not shop at the closest food retailer^(11,12)^. Moreover, transport poverty is influenced by social and geographical factors and is cited as a growing problem in developed countries^(13)^.

Of interest to the current study is Detroit, Michigan, one of the most socially disadvantaged cities in the United States^(14)^ and one of the most segregated metropolitan areas in the country^(15)^. The poverty rate in the city of Detroit is roughly 38 %, with an estimated median household income of roughly $28 000 compared with approximately $53 000 for the state of Michigan^(16)^. Nearly 33 % of households report food insecurity^(17)^ with approximately 41 percent of households enrolled in the Supplemental Nutrition Assistance Program (SNAP)^(17,18)^. Literature has suggested that lack of access to nutritionally dense foods in Detroit minority communities is further hindered by lack of transportation, and approximately 34 % of Detroit residents do not own a vehicle thus relying on the bus system or others for transportation^(19,20)^. Lack of transportation has been identified by Detroit residents as the primary limitation to providing healthy food to their families^(21)^. Moreover, in Detroit, when compared with the most impoverished White neighbourhoods, it has been reported that the most impoverished neighbourhoods, which African Americans resided, were on average 1·1 miles farther from the nearest supermarket^(22)^. Thus, to eradicate food insecurity, research using a critical lens of intersectionality (e.g. race/ethnicity and poverty) is needed to better understand the interaction between the food system within the built environment and the heterogeneity of the population served.

As low-income and African-American populations are more likely to experience food insecurity and are less likely to own a personal vehicle, the objective of the current study was to determine if patients of a large health care system in Detroit who self-identify as food insecure (e.g. screening with Hunger Vital Sign screening tool) live further away from healthy grocery stores (e.g. better Nutrition Environment Measures Survey in Stores; NEM-S scores) compared with food secure patients. We also hypothesised that associations may be reflected in community-level measures of vehicle availability in the area of residence (i.e. perhaps food insecurity is only associated with distance to healthy food stores if they live in areas that have low access to vehicles and typically must walk or take public transit).

## Methods

### Study setting

This is a secondary data analysis that uses baseline data from a pilot intervention/feasibility study (i.e. Henry’s Groceries for Health) conducted through a collaboration between Henry Ford Health System (HFHS) and Gleaners Community Foodbank of Southeastern Michigan (Gleaners). HFHS is a not-for-profit health system based in Detroit, Michigan. It comprises hospitals, medical centres and a large group practice, the Henry Ford Medical Group, which includes more than 1200 physicians practicing in over 40 specialties. HFHS owns Health Alliance Plan, a managed care organisation serving Southeastern Michigan. Gleaners, headquartered in Detroit, links available food to those who need it most by providing nutrition education to households in metro Detroit, operating distribution centres in Wayne, Oakland, Macomb, Livingston and Monroe counties and providing food to 528 partner soup kitchens, food pantries, shelters and other agencies throughout southeast Michigan^(23)^.

### Study sample

**Fig. 1:**
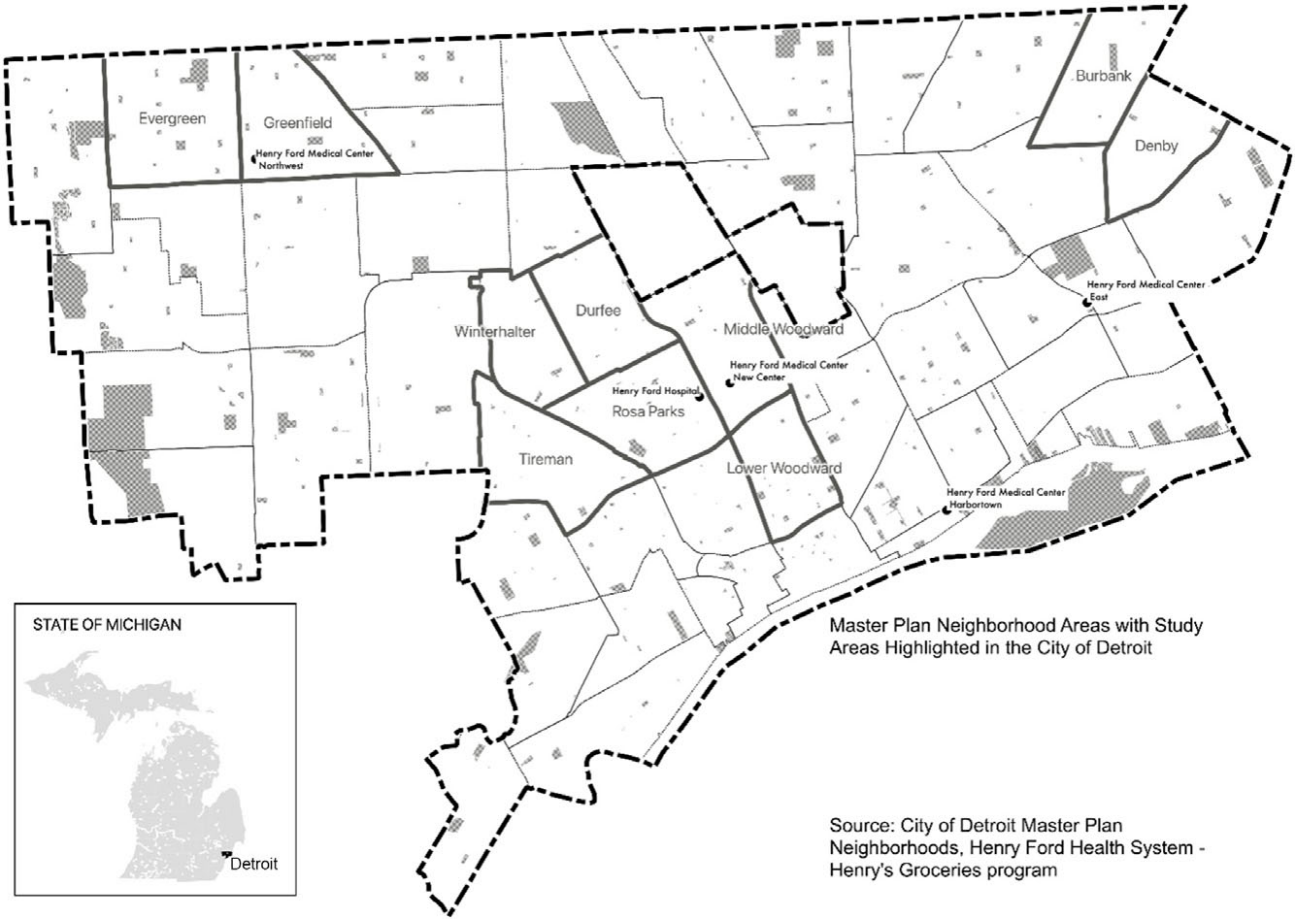
Study area reference map showing counties, city boundary and neighbourhoods

The Henry’s Groceries for Health study screened internal medicine patients >18 years of age who sought medical attention at a participating HFHS clinic between 4 November 2017 and 11 May 2018. Patients included in this secondary data analysis were those who completed the food insecurity screening tool (described below) and resided in an area encompassing Detroit city limits and the cities Highland Park and Hamtramck, both of which are located within Detroit city limits (see Fig. 1 for study area map).

### Dependent variable

#### Food insecurity

Patients were screened by clinic medical assistants using the first two questions in the eighteen-item US Food Security Scale, commonly known as the Hunger Vital Sign screening tool^(24)^ to determine food insecurity. This two-item measure ascertains if within the past 12 months ‘we worried whether our food would run out before we got money to buy more,’ and ‘the food we bought just didn’t last and we didn’t have money to get more.’ Patients were defined as ‘food insecure’ by having answered affirmatively to at least one of the two questions.

### Independent variables

#### Nutrition environment measures survey in stores

The NEMS-S instrument^(25,26)^ consists of eleven measures used to assess the availability, price and quality of healthy foods that are available in retail food stores relative to less healthy choices^(25)^. The NEMS-S instrument includes three sub-scores (i.e. availability, price and quality) as well as a total score (sum of sub-scores). Items from the availability sub-score were assessed on a six-point response scale ranging from 0 to 6. Availability sub-scores for stores can range from 0 to 30, with higher scores representing a greater number of healthier foods available. Items from the price subscale were scored on a four-point Likert-type response scale ranging from −1 to 2 per food item assessed. Price sub-scores for stores can range from −9 to 18 and are derived by comparing the price of the healthier option with the less healthy/regular option, with higher scores representing greater access to healthful foods by both availability and cost. Quality scores of fresh fruit and vegetables were assigned a subjective score ranging from 0 to 6. Subjective ratings were determined by whether greater than 50 % of the item was acceptable (*v.* unacceptable, characterised as rotten, bruised, discoloured or otherwise unappealing). Total NEMS-S score is calculated by summing the sub-scores and can range from −9 to 54.

In the current study, the NEMS-S instrument was administered by the Detroit Food Map Initiative^(27)^ at Wayne State University. A team of trained surveyors have collected data within Detroit’s grocery stores since 2013. All full-line grocery stores (i.e. sells a line of dry groceries, canned goods or nonfood items as well as perishable items such as fresh produce, meat and dairy products) were assessed using NEMS-S. Food Map Initiative in collaboration with the Detroit Economic Growth Corporation and the Detroit Food Policy Council has been tracking store openings and closings in order to maintain an authoritative list. In 2015, a detailed Google Street View assessment was conducted along with field visits to verify grocery store locations^(28)^. A total of 75 full-line grocery stores were assessed and scored within the Detroit city limits, which encompasses and includes the City of Hamtramck and the City of Highland Park.

#### Geocoding and distance to grocery stores

Patient home addresses and NEMS-S scored grocery stores (*n* 75) addresses were geocoded first by using MapInfo Professional 2019 and MapMarker V30 software. The geocoded addresses were then used in ArcGIS Pro V2.4 Network Analyst Extension to calculate and analyse walking and driving distances between each participant’s home and each grocery store (1100 × 75 = 82 500 total distances). For each participant, these distances were then used to calculate the maximum (i.e. ‘healthiest’) and mean (i.e. ‘typical’) NEMS-S score within different walking and driving distances: 0·5, 1 and 2-miles. Presence/absence of any NEMS-S scored grocery stores within these radii was also calculated (i.e. perhaps food insecure patients have no grocery stores around them to begin with, let alone healthy ones). AUC values were also calculated using the trapezoid rule for each participant by plotting the best available NEMS-S score *v*. the distance they would have to travel to reach the store. Large AUC values imply the patient does not have to travel as far to get to a high-scoring grocery store.

### Covariates

Variables such as basic patient demographic information, including sex, race and marital status, were extracted from the electronic medical record. BMI was also extracted from the medical record using the BMI measurement closest to – but not after – the day of study screening. Additional socio-demographic covariates were retrieved at the census tract level based on geocoded address, for both patients and grocery store locations. These data were pulled from the 2014–2018 American Community Survey conducted by the United States Census Bureau and included median household income, income to poverty ratio, unemployment rate, uninsured rate, percentage without a vehicle and percentage with public assistance or food stamps/SNAP.

### Statistical analyses

All analyses were performed using R version 3.5.2 (2018–12–20). The threshold for statistical significance was prespecified at *P* < 0·05. Food insecure and food secure patients were first compared by demographic characteristics using independent samples *t*-test for continuous covariates and the *χ* ^2^ test for categorical covariates. To examine census-tract-level covariates associated with grocery store NEMS-S scores, Pearson correlation coefficients were calculated.

To associate NEMS-S score variables with food insecurity, multiple imputation was first performed using the R package *mice* due to a high rate of missingness in the EMR-abstracted covariates; five random forest imputations were calculated. Food insecurity and all covariates were included in the imputation algorithm, as well as whether an NEMS-S scored grocery store existed within 0·5, 1 and 2-mile radii.

Once the imputed data sets were calculated, they were used in linear regression models to associate NEMS-S score and AUC values with food insecurity, and estimates were pooled across imputations. NEMS sub-scores were also associated with food insecurity similarly, though quality could not be examined separately due to lack of variability (72 of the 75 stores had a quality score of 6). Models were fit both unadjusted and adjusted for all descriptive covariates significantly associated with food insecurity (*P* < 0·05). Similarly, logistic regression was used to associate presence/absence of any NEMS-S scored grocery stores within a 0·5 and 1 mile radius with food insecurity; 2-mile radius was not examined in logistic regression analyses due to small sample sizes (only seventeen patients did not have a store within 2-miles of their home). For all outcomes, effect modification by census tract vehicle availability was tested using interaction terms; subgroup-specific effects were also examined. We considered areas with a high percentage of residents without vehicles to be the third quartile or more (≥17 %).

In order to identify potential ‘hotspots’ of food insecurity, or sub-regions of Detroit where the risk of food insecurity is significantly elevated, spatial relative risk based on the ratio of two kernel densities were estimated using the R package *sparr*, using the symmetric case adaptive method^(29)^.

## Results

### Description of food insecure and food secure patients

A total of 1100 patients were included in the analysis, 336 (31 %) of which were food insecure. Patients who were food insecure tended to be younger at screening (*P* < 0·001) and were less likely to be married (*P* < 0·001) according to data collected from the EMR (Table 1). Using community-level data, persons reporting food insecurity also tended to live in areas with: lower median household incomes (*P* = 0·014), higher percentages of people with income less than the poverty rate (*P* = 0·019), higher unemployment rates (*P* = 0·014), higher rates of no vehicle access (*P* = 0·022) and higher rates of public assistance or food stamps/SNAP (*P* = 0·014).

**Table 1:** Comparison of food insecure and food secure patients included in analysis (*n* 1100)

	Food secure		Food insecure		*P*-value	*n*
	*n*	%	*n*	%		
	*n 764*		*n 336*			
Age at screening						
Mean	67·3		59·1		<0·001	1085
sd	16·1		14·1			
Sex					0·285	905
Female	358	62·6 %	221	66·4 %		
Male	214	37·4 %	112	33·6 %		
Race					0·338	856
Black	507	94·2 %	294	92·5 %		
White	21	3·90 %	13	4·09 %		
Mixed/Other	10	1·86 %	11	3·46 %		
Married					<0·001	892
No	339	60·0 %	251	76·8 %		
Yes	226	40·0 %	76	23·2 %		
BMI						
Mean	31·3		32·6		0·053	846
sd	7·95		9·78			
Median household income						
Mean	30 445		28 420		0·014	1100
sd	13 455		12 195			
Percent with income less than poverty level						
Mean	30·5		32·5		0·019	1100
sd	13·4		13·1			
Percent unemployed						
Mean	17·6		18·8		0·014	1100
sd	7·44		7·58			
Percent uninsured						
Mean	8·99		8·62		0·134	1100
sd	3·81		3·78			
Percent without a vehicle						
Mean	12·8		14·4		0·022	1100
sd	10·7		11·1			
Percent with public assistance or food stamps/SNAP						
Mean	41·0		43·0		0·014	1100
sd	12·7		12·7			

### Description of NEMS-S scored grocery stores

Initial descriptives revealed that walking and driving distances produced identical scores and were therefore used interchangeably in analysis. The average NEMS-S score among the 75 grocery stores within Detroit city limits was 27 (sd = 5, Min = 16, Max = 35). When census-tract-level variables were examined for an association with NEMS-S score, only median household income was significantly associated with NEMS-S score, whereby stores located in areas with higher median household incomes tended to have higher NEMS-S scores (*r* = 0·24, *P* = 0·043). Percent unemployment was also marginally negatively correlated with NEMS-S score (*r* = −0·19, *P* = 0·098). Percent with income less than poverty level (*r* = −0·15, *P* = 0·20), percent without a vehicle (*r* = 0·07, *P* = 0·55), percent uninsured (*r* = 0·04, *P* = 0·74) and percent with public assistance or food stamps/SNAP (*r* = −0·19, *P* = 0·11) did not significantly associate with NEMS-S score.

### Associating food insecurity with distance to healthy food source

When NEMS-S score was examined in relation to food insecurity (Table 2), no unadjusted associations reached statistical significance. However, after covariate adjustment, the maximum NEMS-S score in a 2-mile radius significantly associated with food insecurity, where food insecure patients on average had 0·39 higher NEMS-S scores. No other models reached significance, but the direction of association was consistent throughout. Further, results were consistent when NEMS sub-scores were examined (Table 2). Specifically, after covariate adjustment, the maximum price score in a 1-mile radius was 0·40 higher among food insecure patients (*P* = −0·025), while the average price score in a 1-mile radius was 0·43 higher (*P* = 0·007), and the maximum availability score in a 2-mile radius was 0·24 higher (*P* = 0·037). Among food insecure patients residing in areas of higher vehicle ownership, total NEMS-S scores were concomitantly higher (Table 3). However, none of these interaction p-values were statistically significant (all interaction *P* ≥ 0·33).

**Table 2:** Association between food insecurity and NEMS score

Outcome	Model	*n*	Estimate*	se	Lower 95 % CI	Upper 95 % CI	*P*-value
Max NEMS in a 0·5 mi radius	Unadjusted	273	0·05	0·57	−1·07	1·18	0·926
Max NEMS in a 0·5 mi radius	Adjusted	273	0·24	0·55	−0·84	1·33	0·659
Mean NEMS in a 0·5 mi radius	Unadjusted	273	0·07	0·57	−1·06	1·20	0·905
Mean NEMS in a 0·5 mi radius	Adjusted	273	0·31	0·55	−0·77	1·39	0·569
Max NEMS in a 1 mi radius	Unadjusted	791	0·44	0·32	−0·19	1·07	0·169
Max NEMS in a 1 mi radius	Adjusted	791	0·48	0·32	−0·15	1·11	0·138
Mean NEMS in a 1 mi radius	Unadjusted	791	0·53	0·32	−0·10	1·15	0·098
Mean NEMS in a 1 mi radius	Adjusted	791	0·62	0·32	−0·01	1·24	0·053
Max NEMS in a 2 mi radius	Unadjusted	1083	0·27	0·19	−0·11	0·64	0·161
Max NEMS in a 2 mi radius	Adjusted	1083	0·39	0·19	0·02	0·76	0·039
Mean NEMS in a 2 mi radius	Unadjusted	1083	0·14	0·18	−0·22	0·49	0·447
Mean NEMS in a 2 mi radius	Adjusted	1083	0·28	0·18	−0·07	0·62	0·114
Max price score in a 0·5 mi radius	Unadjusted	273	0·43	0·31	−0·18	1·04	0·165
Max price score in a 0·5 mi radius	Adjusted	273	0·47	0·31	−0·13	1·07	0·127
Max availability score in a 0·5 mi radius	Unadjusted	273	−0·32	0·39	−1·09	0·44	0·406
Max availability score in a 0·5 mi radius	Adjusted	273	−0·24	0·39	−1·01	0·53	0·542
Mean price score in a 0·5 mi radius	Unadjusted	273	0·45	0·31	−0·16	1·06	0·144
Mean price score in a 0·5 mi radius	Adjusted	273	0·53	0·31	−0·08	1·13	0·086
Mean availability score in a 0·5 mi radius	Unadjusted	273	−0·33	0·39	−1·09	0·44	0·403
Mean availability score in a 0·5 mi radius	Adjusted	273	−0·15	0·39	−0·92	0·61	0·692
Max price score in a 1 mi radius	Unadjusted	791	0·44	0·17	0·10	0·77	0·012
Max price score in a 1 mi radius	Adjusted	791	0·40	0·18	0·05	0·74	0·025
Max availability score in a 1 mi radius	Unadjusted	791	−0·01	0·23	−0·45	0·43	0·963
Max availability score in a 1 mi radius	Adjusted	791	0·01	0·23	−0·44	0·46	0·96
Mean price score in a 1 mi radius	Unadjusted	791	0·47	0·16	0·16	0·78	0·003
Mean price score in a 1 mi radius	Adjusted	791	0·43	0·16	0·12	0·75	0·007
Mean availability score in a 1 mi radius	Unadjusted	791	0·04	0·23	−0·41	0·48	0·875
Mean availability score in a 1 mi radius	Adjusted	791	0·14	0·23	−0·31	0·59	0·538
Max price score in a 2 mi radius	Unadjusted	1083	0·16	0·13	−0·09	0·41	0·214
Max price score in a 2 mi radius	Adjusted	1083	0·19	0·13	−0·06	0·44	0·132
Max availability score in a 2 mi radius	Unadjusted	1083	0·16	0·11	−0·06	0·39	0·145
Max availability score in a 2 mi radius	Adjusted	1083	0·24	0·11	0·01	0·46	0·037
Mean price score in a 2 mi radius	Unadjusted	1083	0·05	0·08	−0·11	0·21	0·512
Mean price score in a 2 mi radius	Adjusted	1083	0·08	0·08	−0·08	0·24	0·346
Mean availability score in a 2 mi radius	Unadjusted	1083	0·09	0·12	−0·15	0·34	0·455
Mean availability score in a 2 mi radius	Adjusted	1083	0·20	0·12	−0·04	0·44	0·097

* Estimates are interpreted as the mean difference in NEMS sub-score comparing food insecure with food secure patients. Adjusted models include age, marital status, median household income, percent with income less than poverty level, unemployment rate, vehicle availability rate and public assistance/food stamps usage.

**Table 3:** Association between food insecurity and NEMS score, by vehicle availability

Outcome	Interaction *P*-value	Subgroup	*n*	Estimate*	Lower 95 % CI	Upper 95 % CI	*P*-value
Max NEMS in a 0·5 mi radius	0·509	Low % without vehicle	207	0·62	−0·77	2·01	0·380
Max NEMS in a 0·5 mi radius		High % without vehicle	66	−0·39	−2·26	1·48	0·678
Mean NEMS in a 0·5 mi radius	0·571	Low % without vehicle	207	0·70	−0·69	2·09	0·321
Mean NEMS in a 0·5 mi radius		High % without vehicle	66	−0·30	−2·18	1·59	0·754
Max NEMS in a 1 mi radius	0·97	Low % without vehicle	583	0·58	−0·19	1·35	0·138
Max NEMS in a 1 mi radius		High % without vehicle	208	0·31	−0·80	1·41	0·585
Mean NEMS in a 1 mi radius	0·855	Low % without vehicle	583	0·74	−0·02	1·50	0·058
Mean NEMS in a 1 mi radius		High % without vehicle	208	0·51	−0·58	1·61	0·355
Max NEMS in a 2 mi radius	0·653	Low % without vehicle	795	0·54	0·09	0·99	0·019
Max NEMS in a 2 mi radius		High % without vehicle	288	0·08	−0·55	0·72	0·794
Mean NEMS in a 2 mi radius	0·331	Low % without vehicle	795	0·50	0·10	0·90	0·014
Mean NEMS in a 2 mi radius		High % without vehicle	288	−0·16	−0·86	0·54	0·657

* Estimates are interpreted as the mean difference in NEMS score comparing food insecure with food secure patients, within specified subgroup. Models are adjusted for age, marital status, median household income, percent with income less than poverty level, unemployment rate and public assistance/food stamps usage.

In models evaluating the association between the presence of an NEMS-S scored grocery store at different distances and food insecurity (Table 4), no significant associations were found. When these associations were evaluated by vehicle availability (Table 5), the association between food insecurity and having a full-line grocery store within a 1-mile radius was found to significantly differ depending on vehicle availability in the area of residence (interaction *P* = 0·045). Specifically, food insecure patients had 1·64 times higher odds of living within 1-mile of any NEMS-S scored grocery store, but only among those who lived in areas which has a high percentage of residents without a vehicle. However, this subgroup-specific effect failed to reach statistical significance (*P* = 0·107).

**Table 4:** Association between food insecurity and existence of an NEMS-scored grocery store

Outcome	Model	*n*	OR*	Lower 95 % CI	Upper 95 % CI	*P*-value
NEMS-scored grocery store within in a 0·5-mile radius	Unadjusted	1100	1·06	0·79	1·43	0·692
NEMS-scored grocery store within in a 0·5-mile radius	Adjusted	1100	0·98	0·72	1·33	0·887
NEMS-scored grocery store within in a 1-mile radius	Unadjusted	1100	1·10	0·82	1·47	0·523
NEMS-scored grocery store within in a 1-mile radius	Adjusted	1100	1·05	0·77	1·41	0·770

* OR are interpreted as the increase in the odds of an NEMS-scored grocery store being present in an X-mile radius, comparing food insecure with food secure patients. Adjusted models include age, marital status, median household income, percent with income less than poverty level, unemployment rate, vehicle availability rate and public assistance/food stamps usage.

**Table 5:** Association between food insecurity and existence of an NEMS-scored grocery store, by vehicle availability

Outcome	Interaction *P*-value	Subgroup	*n*	OR*	Lower 95 % CI	Upper 95 % CI	*P*-value
NEMS-scored grocery store within in a 0·5-mile radius	0·615	Low % without vehicle	812	0·97	0·67	1·39	0·848
NEMS-scored grocery store within in a 0·5-mile radius		High % without vehicle	288	1·12	0·62	2·03	0·707
NEMS-scored grocery store within in a 1-mile radius	0·045	Low % without vehicle	812	0·89	0·62	1·26	0·512
NEMS-scored grocery store within in a 1-mile radius		High % without vehicle	288	1·64	0·90	2·99	0·107

* OR are interpreted as the increase in the odds of an NEMS-scored grocery store being present in an X-mile radius, comparing food insecure with food secure patients, within specified subgroup. Models are adjusted for age, marital status, median household income, percent with income less than poverty level, unemployment rate and public assistance/food stamps usage.

We next evaluated the AUC values (mean = 479, sd = 5·3), where larger values suggest that a shorter distance is required to get to a high NEMS-S scoring grocery store. Food insecure patients on average had higher AUC values (i.e. shorter distances to higher-scoring stores), consistent with previous results of the current study (Table 6). However, this association did not reach statistical significance both before and after covariate adjustment. When effect modification by vehicle access was examined (Table 7), a significant interaction was not identified (*P* = 0·077).

**Table 6:** Association between food insecurity and AUC

Outcome	Model	*n*	Estimate*	se	Lower 95 % CI	Upper 95 % CI	*P*-value
AUC	Unadjusted	1100	0·11	0·34	−0·56	0·79	0·739
AUC	Adjusted	1100	0·23	0·34	−0·44	0·90	0·502

* Estimates are interpreted as the mean difference in AUC comparing food insecure with food secure patients. Adjusted models include age, marital status, median household income, percent with income less than poverty level, unemployment rate, vehicle availability rate and public assistance/food stamps usage.

**Table 7:** Association between food insecurity and AUC by vehicle availability

Outcome	Interaction *P*-value	Subgroup	*n*	Estimate*	Lower 95 % CI	Upper 95 % CI	*P*-value
AUC	0·077	Low % Without Vehicle	812	0·84	0·07	1·60	0·032
AUC		High % Without Vehicle	288	−1·05	−2·42	0·32	0·133

* Estimates are interpreted as the mean difference in AUC comparing food insecure with food secure patients, within specified subgroup. Models are adjusted for age, marital status, median household income, percent with income less than poverty level, unemployment rate and public assistance/food stamps usage.

### Mapping patients by food insecurity status

**Fig. 2:**
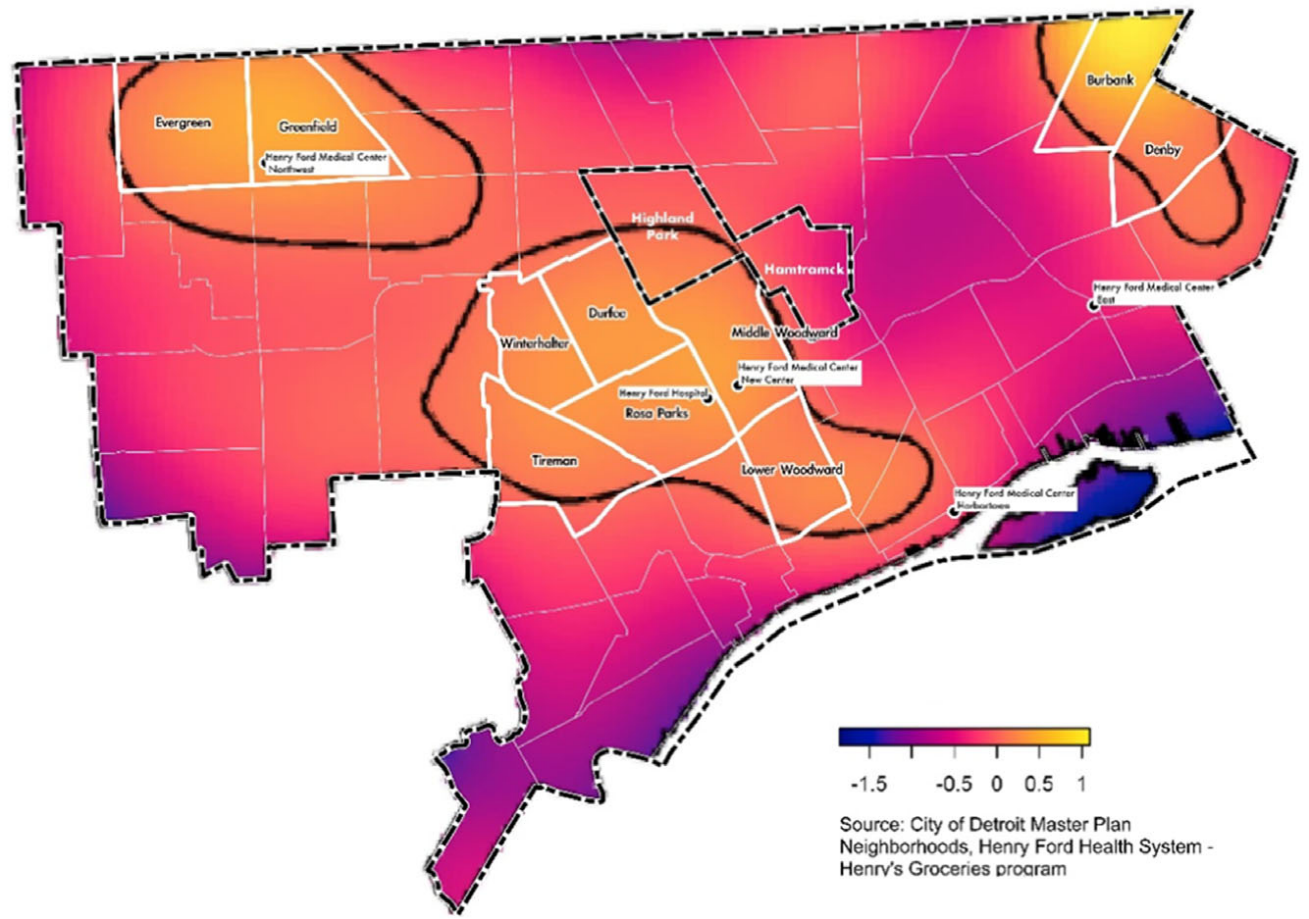
Estimation of spatial relative risk (on the log scale) of food insecurity using four different estimation algorithms. Larger values indicate increased risk of food insecurity. Contour lines indicate sub-regions of significantly elevated risk at level 0·05

When the spatial relative risk of food insecurity was mapped (Fig. 2), a significantly increased risk of food insecurity was found in the centre of Detroit city limits, which were roughly south of Highland Park and west of Hamtramck (Middle Woodward, also known as New Center/North End). Additional high-risk areas included the city of Highland Park, as well as the City of Detroit Master Plan of Policies (i.e. official local government planning which identifies geographic organisation and boundaries), neighbourhood areas of Rosa Parks, Durfee and Middle Woodward in Central Detroit, Burbank and Denby in Northeast (also known as Osborn and Regent Park) and Evergreen/Greenfield area of Northwest Detroit. These neighbourhoods are generally composed of a high percentage of minorities and have low mean per capita incomes, high rates of poverty and low vehicle access (Table 8).

**Table 8:** Demographics of Detroit neighbourhoods having a signficantly increased risk of food insecurity

	Per capita income (mean)	Poverty (mean)	Minority status (mean)	No vehicle access (mean)
Detroit	$16 858	28·0 %	88·6 %	16·2 %
Central (Rosa Parks/Durfee/Winterhalter/Tireman/Middle Woodward/Lower Woodward)	$16 584	22·4 %	88·6 %	13·1 %
Northeast (Burbank/Denby)	$15 187	34·2 %	97·2 %	17·0 %
Northwest (Evergreen/Greenfield)	$17 732	34·1 %	97·7 %	20·6 %

## Discussion

The overall goal of the current study was to better understand where food insecure patients in Detroit reside, and if they live further away from healthy grocery stores compared with food secure patients. Based on literature surrounding the intersectionality of race/ethnicity and poverty as well as the inequalities described by transport poverty^(19,20,22)^, it had been hypothesised in the current study that associations between food insecurity and access to healthy grocery stores may depend upon community-level measures of vehicle availability in the area of residence. After accounting for socio-demographic factors associated with food insecurity, we did not find evidence that food insecure patients lived further away from healthier grocery stores, nor was this modified by ecological measures of vehicle access. However, neighbourhoods with more patients who identified as food insecure tended to be areas with high rates of poverty and a large percentage of minorities, suggesting that structural and systemic biases in the food environment may also be present.

The characteristics and demographics associated with food insecurity in the current study are similar to those presented in previous research (e.g. households with low levels of income, minorities)^(5)^, and this is particularly true in the highlight ‘hotspot’ neighbourhoods found within the Detroit city limits. In regard to proximity to healthy grocery stores, although contrary to our original hypothesis, findings of the current study compliment those which have recently emerged in the literature^(30–33)^, emphasising the need to consider whether those identifying as food insecure, actually utilise the food stores around them and the effects of contextual factors (e.g. economic access, capacity to cope with risk, food provision, etc.)^(34)^ that should be accounted for whilst attempting to understand food insecurity^(35–37)^. Some literature^(36,38)^ has shown that individuals routinely conduct day-to-day activities (e.g. work, child care, social engagements and shopping) outside their residential neighbourhood, which could have implications on the findings of the current study^(30,38)^. In addition, studies have found that most low-income individuals, including food insecure individuals, travel outside of their neighbourhoods to shop^(30,39)^. It is essential for studies to investigate where people shop, with or for whom, and why, rather than assuming individuals choose to minimise distance in their shopping preferences^(35,40)^.

Detroiters that identify as food insecure may be living in a what has been coined in the literature as a ‘food mirage’ rather than a ‘food desert’^(41)^. Food mirages have been described as neighbourhoods appearing to have adequate food access but that for some minority residents and those with less education and income find the grocery stores to be too expensive or culturally unfamiliar^(42,43)^. Future research should consider using the 5 A’s of Food Access (access, availability, affordability, accommodation and acceptability)^(44)^ as a theoretical model during study design. The current study did not account for if the food retailers accommodate (e.g. convenient hours, forms of payment) the population served (accommodation) or whether the population wants to buy and eat the food that is being sold (acceptability). Acceptability is defined by Rocha^(45)^, as ‘food that is culturally acceptable, produced and obtained in ways that do not compromise people’s dignity, self-respect and human rights’. There is a need to provide a more robust picture of the food environment when exploring food insecurity. Within society there is a tendency to build more, do more and impose more structures but perhaps these efforts are misguided. Future studies should aim to explore barriers and contextual factors to change such as the complex coping strategies low-income individuals use to acquire foods that meet their needs and preferences (e.g. visiting multiple stores to get the best deals and maximise their food dollars)^(46–48)^ and how household dynamics (e.g. nutritional knowledge)^(35)^ influence respondents’ interactions with food sources.

### Limitations

Several limitations of the current study need to be acknowledged. The current study NEMS-S scores only reflect grocery stores. Focusing solely or primarily on full-line grocery stores misses other sources from which people can obtain food in the city. Moreover, information on primary grocery store utilisation was not collected from participants. Second, some patient covariates (e.g. vehicle ownership, median household income) were measured at the census tract level rather than directly reported by the patient. These associations, while generally in the direction reported by previous studies, are subject to the ‘ecological fallacy’ and should be considered exploratory^(49)^. Finally, a limitation of the secondary analysis is that it was not possible to ask further explanatory questions that might have clarified the data. However, the current study has important strengths including the definition of food insecurity used, which was based on a validated two-item screener that has been shown to have good sensitivity, specificity and validity. Additionally, the quality of grocery stores was systematically quantified using the NEMS-S, which has a high degree of inter-rater and test–retest reliability^(25)^. Moreover, city wide NEMS-S have not been readily conducted. Of those few cities (e.g. Baltimore^(50)^, New Haven^(51)^, Detroit^(39)^ and Flint^(26)^) that do have this measure available, a modified version of NEMS-S was used in publication, making it difficult to compare findings without access to the complete data sets for other cities.

## Conclusion

The relationship between being low-income and a racial minority and its connection to food insecurity is complex. It appears food insecure patients in Detroit are perhaps limited by contextual factors and not their immediate neighbourhood or physical access to healthy grocery stores. Future research should explore the complexity in linkages between household socio-economic factors, socio-cultural dynamics and the neighbourhood food environment.
